# The Role of Para-Aortic Lymphadenectomy in the Surgical Staging of Women with Intermediate and High-Risk Endometrial Adenocarcinomas

**DOI:** 10.1155/2013/858916

**Published:** 2013-02-27

**Authors:** Taymaa May, Melina Shoni, Allison F. Vitonis, Charles M. Quick, Whitfield B. Growdon, Michael G. Muto

**Affiliations:** ^1^Department of Obstetrics and Gynecology, Division of Gynecologic Oncology, Brigham and Women's Hospital, Harvard Medical School, Boston, MA 02115, USA; ^2^Division of Gynecologic Oncology, Dana-Farber Cancer Institute, Boston, MA 02115, USA; ^3^Department of Obstetrics and Gynecology, Epidemiology Center, Brigham and Women's Hospital, Boston, MA 02115, USA; ^4^Department of Pathology, University of Arkansas for Medical Sciences, Little Rock, AR 72205, USA; ^5^Department of Obstetrics and Gynecology, Division of Gynecologic Oncology, Massachusetts General Hospital, Harvard Medical School, Boston, MA 02214, USA

## Abstract

*Objectives*. To characterize clinical outcomes in patients with intermediate or high-risk endometrial carcinoma who underwent surgical staging with or without para-aortic lymphadenectomy. *Methods*. This is a retrospective cohort study of patients with intermediate or high-risk endometrial adenocarcinoma who underwent surgical staging with (PPALN group) or without (PLN) para-aortic lymphadenectomy. Data were collected, Kaplan-Meier curves were generated, and univariate and multivariate analyses performed to compare differences in adjuvant therapy, disease recurrence, disease-free survival (DFS), and overall survival (OS). *Results.* 118 patients were included in the PPALN group and 139 in the PLN group. Patients in the PPALN group were more likely to receive adjuvant vaginal brachytherapy (25.4% versus 11.5%, OR = 2.5, *P* = 0.03) and less likely to receive adjuvant multimodal combination therapy (17.81% versus 28.8%, OR = 0.28, *P* = 0.002). DFS was improved in the PLN group as compared to PPALN (80% versus 62%, *P* = 0.02). OS was equivalent (*P* = 0.93). Patients in the PPALN group who had less than 10 para-aortic nodes removed were twice as likely to recur than patients who had 10 or more para-aortic nodes or patients in the PLN group (HR 2.08, CI 1.20–3.60, *P* = 0.009). *Conclusions*. Patients in the PLN group were more likely to receive multimodal adjuvant therapy and had better DFS than the PPALN group. Pelvic lymphadenectomy followed by adjuvant radiation and chemotherapy may represent an effective treatment option for patients with intermediate or high-risk disease. If systematic para-aortic lymphadenectomy is performed and less than 10 para-aortic lymph nodes are obtained, multimodality adjuvant therapy should be considered to improve DFS.

## 1. Introduction

The landmark study GOG 33 described the patterns of spread in endometrial carcinoma and concluded that clinical staging is inaccurate as 22% of clinical stage I patients were assigned a higher surgical stage [1]. As such, the International Federation of Gynecology and Obstetrics (FIGO) changed the endometrial cancer staging system from clinical to surgical [2]. Conventionally, surgical staging includes a total hysterectomy, bilateral salpingooophorectomy, and retroperitoneal pelvic and para-aortic lymphadenectomy. Although pelvic washings are no longer part of the 2009 FIGO surgical staging system, they are still collected at time of surgery [2]. 

Multivariate analysis of GOG 33 indicated 3 uterine factors as independent predictors of nodal metastasis, including tumor grade, depth of myometrial invasion, and the presence of intraperitoneal disease [3]. Using these factors as predictors of disease aggressive behavior, endometrial carcinoma is often divided into low, intermediate, and high-risk diseases [3]. Typically, patients with intermediate and high-risk diseases undergo surgical staging. However, the beneficial effect of complete, systematic lymphadenectomy is debatable. Several studies reported increased morbidity associated with the addition of retroperitoneal lymphadenectomy to the surgical procedure including increased mean blood loss, increased risk of blood transfusion, increased operative time and longer hospital stay [4, 5]. Additionally, lymphadenectomy increases the risk of postoperative fever, incision site infection, lymphocyst formation, lower-extremity edema, embolic events, gastrointestinal obstruction, and perioperative mortality [6]. Notably, the addition of para-aortic lymph node dissection further increases the surgical morbidity. Cragun et al. reported increased blood loss, transfusion rates, and length of hospital stay in patients undergoing both pelvic and para-aortic lymphadenectomy as compared to patients undergoing pelvic lymphadenectomy alone [7]. 

We designed a study examining the role of para-aortic lymphadenectomy in the surgical staging of patients with intermediate and high-risk endometrial adenocarcinomas. Our objectives were to assess whether or not para-aortic lymphadenectomy impacts administration of adjuvant therapy, disease recurrence, disease-free survival (DFS), and overall survival (OS).

## 2. Materials and Methods

### 2.1. Study Design

This a retrospective cohort study investigating patients who underwent surgical staging for newly diagnosed high-grade endometrioid, serous, or clear cell endometrial adenocarcinoma at Brigham and Women's Hospital and Massachusetts General Hospital, Harvard Medical School, Boston, MA, USA, between January 2000 and December 2010. Institutional review board (IRB) approval was obtained from the hospitals' ethics board. Eligible patients were identified using the hospitals' pathology data base and data points were obtained from the patients' electronic medical records. 

### 2.2. Study Population

The first study group included patients who underwent primary surgical staging including total abdominal, laparoscopic or robotic hysterectomy, bilateral salpingooophorectomy, washings, and pelvic and para-aortic lymphadenectomy (PPALN group). The second study group included patients who underwent a similar staging procedure with the exception of the para-aortic lymphadenectomy (PLN group). Data were collected from the patients' hospital charts and analyzed using appropriate statistical tests. 

### 2.3. Outcome Measures

The primary outcome measure of this study was to compare overall survival (OS) between the two study groups to evaluate the impact that para-aortic lymphadenectomy has on OS. The secondary outcome measures were to examine whether the absence of a para-aortic lymphadenectomy impacts administration of adjuvant therapy, disease recurrence, or disease-free survival (DFS). 

### 2.4. Statistical Analysis

Chi-square, Fisher's exact tests, and *t*-tests were used to compare the characteristics of patients in the two study groups. Kaplan-Meier curves and Cox proportional hazards models were used to compare OS and DFS between the groups. Models were adjusted for age, year of surgery, histology, lymphovascular invasion, myometrial invasion, and adjuvant therapy.All analyses were performed using SAS version 9.2 (SAS Institute Inc., Cary, NC, USA).

## 3. Results

### 3.1. Population Characteristics

 Of all women diagnosed with endometrial carcinoma at Brigham and Women's Hospital and Massachusetts General Hospital, Boston, MA, USA, between January 2000 and December 2010, 257 met our inclusion criteria and were subjected to our final analysis. The PPALN group was composed of 118 patients, while 139 patients underwent PLN. The mean age at time of diagnosis in the PPALN group was 63.1, and in the PLN group it was 67.1 (*P* = 0.002). Importantly, survival was not significantly altered when controlling for the difference in age. Demographic and clinical characteristics of the study cohort are provided in Table 1. 

### 3.2. Clinical and Surgical Characteristics

The surgical stages were similar between the PPALN group and the PLN group (Table 1). Patients in the PLN group had higher rates of papillary serous histology (32.4% versus 19.7%, *P* = 0.02) and lower rates of grade 3 endometrioid carcinoma (23.7% versus 44.4%) than patients in the PPALN group. Risks of recurrence and DFS were not affected when controlling for the differences in histology using multivariate analysis (Table 2). The other histological subtypes were similar between the two groups. Patients in the PPALN group had significantly higher lymphovascular space invasion (52.3% versus 35.2%, *P* = 0.008) and higher outer half myometrial invasion (47.4% versus 35.3%, *P* = 0.05). Risks of recurrence and DFS were not significantly affected when controlling for these variables by multivariate analysis (Table 2). The intraoperative complications studied included cystotomy, enterotomy, vascular injury, ureteral injury, and intraoperative blood transfusion. Postoperative complications studied included fever, blood transfusion, paralytic ileus, small bowel obstruction, wound cellulitis, deep wound infection, and reoperation within 28 days of original surgery. Intraoperative and postoperative complication rates were equivalent between the groups (*P* = 0.36 and *P* = 0.09, resp.). The mean number of pelvic nodes removed per patient in the PLN group was 10.7 (range 1–35). The mean numbers of pelvic and para-aortic nodes in the PPALN group were 16.1 (range 2–40) and 5.3 (range 1–19), respectively. Forty-one patients (29.4%) in the PLN group had positive pelvic lymph nodes (Table 3). In the PPALN group, 34 patients (28.8%) had positive pelvic lymph nodes, and 26 patients (22.03%) had positive para-aortic lymph nodes. Of the 26 patients with positive para-aortic lymph nodes, 20 (16.9%) had concurrent positive pelvic lymph nodes, and 6 (5.08%) had negative pelvic lymph nodes (Table 3). 

### 3.3. Treatment and Recurrence

**Figure 1:**
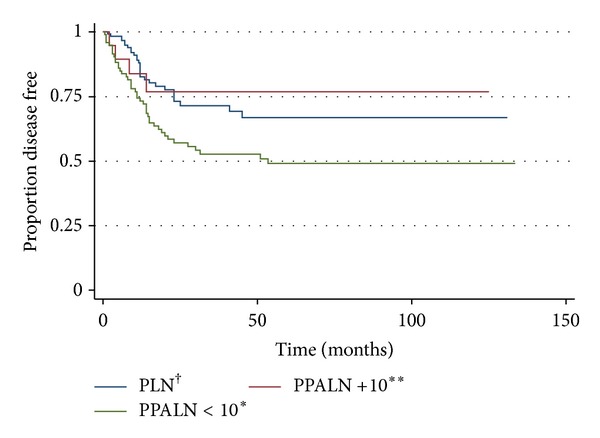
Kaplan-Meier disease-free survival estimate. PPALN < 10* versus PLN^†^ or PPALN+10** logrank test: HR 2.34, CI 1.36–4.02, *P* = 0.002. *Patients in the pelvic and para-aortic Lymph node (PPALN) group with less than 10 para-aortic lymph nodes retrieved at time of dissection. **Patients in the pelvic and para-aortic lymph node (PPALN) group with 10 or more para-aortic lymph nodes retrieved at time of dissection. ^†^Patients in the pelvic lymph node (PLN) group.

Patients in the PPALN group were more likely to receive adjuvant vaginal brachytherapy (25.4% versus 11.5%, OR = 2.5, *P* = 0.03) and less likely to receive adjuvant multimodal therapy consisting of combined vaginal brachytherapy, pelvic radiation and chemotherapy (17.8% versus 28.8%, OR = 0.28, *P* = 0.0019) (see Table  1(a) in Supplementary Material available online at http://dx.doi.org/10.1155/2013/858916). Patients in the PPALN group were more likely to experience disease recurrence than patients in the PLN group (38.9% versus 20.14%, *P* = 0.003). Variation in adjuvant therapy was not an independent predictor of recurrence, DFS or OS (see Tables  1(b) and  1(c) in Supplementary Material). The number of para-aortic nodes removed at time of surgery was associated with disease recurrence. Patients in the PPALN group who had less than 10 para-aortic nodes removed were twice more likely to recur than patients who had 10 or more para-aortic nodes or patients in the PLN group (HR 2.34, CI 1.36–4.02, *P* = 0.002) (Figure 1). As such, the number of para-aortic lymph nodes obtained at time of surgery was an independent factor associated with disease recurrence and DFS (Table 2). Abdominal recurrences represented a significantly increased portion of recurrences in the PLN group compared to the PPALN group (53.6% versus 28.3%, *P* = 0.03) (Table 4(a)). Recurrence patterns at other sites including vagina, pelvis, pelvic lymph nodes, para-aortic lymph nodes and extra-peritoneal sites were similar between the groups (Table 4(a)). Cox proportional hazards model for overall survival showed no association between recurrence site and survival (Table 4(b)). These analyses were adjusted for age (continuous), year of surgery (continuous), lymph nodes (PLN and PALN), histology (endometrioid, mixed, clear cell, and papillary serous), lymphovascular invasion, and myometrial invasion.

### 3.4. Disease Free and Overall Survival

**Figure 2:**
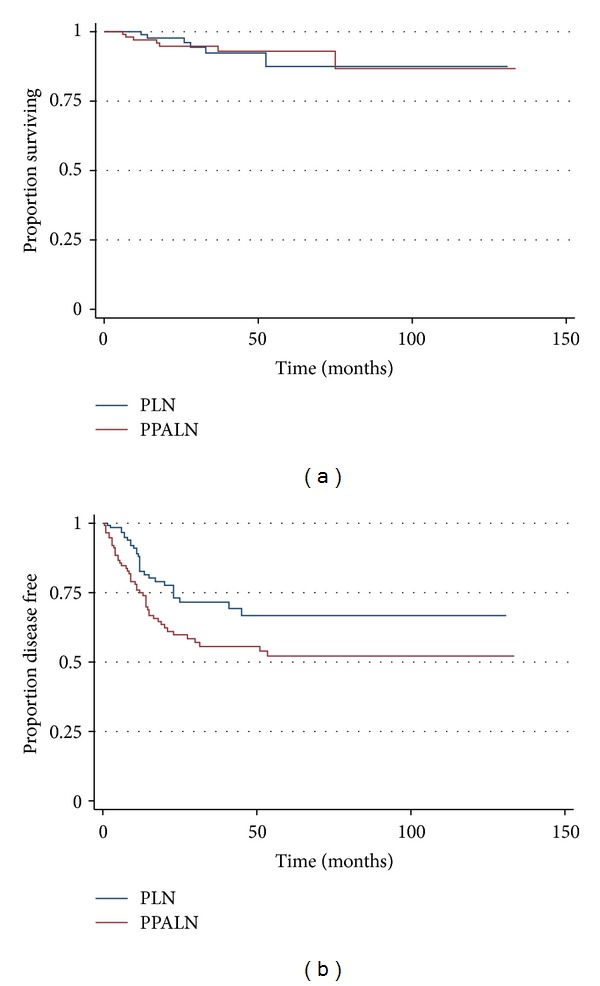
(a) Kaplan-Meier overall survival estimate. Logrank test PLN versus PPALN *P* value = 0.93. (b) Kaplan-Meier Disease-Free survival estimate. PLN versus PPALN Logrank test *P* value = 0.02.

OS was similar between the PLN and the PPALN groups (*P* = 0.93) (Figure 2(a)). Patients in the PLN group had better DFS than patients in the PPALN group (80% versus 62%, *P* = 0.02) (Figure 2(b)). The mean followup time was 32.4 months.

## 4. Discussion

Our study investigates the role and extent of retroperitoneal lymphadenectomy in the management of women with intermediate and high-risk endometrial adenocarcinomas. Women who underwent para-aortic lymph node dissections had an overrepresentation of deep myometrial invasion, lymphovascular invasion, and grade 3 endometrioid histology, and they were less likely to undergo postoperative multimodality adjuvant therapy. Cox proportional hazards models as well as multivariate analysis were adjusted for age, year of surgery, histology, lymphovascular invasion, myometrial invasion and adjuvant therapy to control for the variations within the groups. Multivariate analysis incorporating these significant variables along with the extent of lymphadenectomy confirmed that only para-aortic lymphadenectomy yielding less than 10 nodes was associated with an increased risk of recurrence and decreased PFS. No difference in OS was observed between the groups. These data suggest that limited para-aortic lymph node dissection may not obviate the need for aggressive, multimodality adjuvant therapy based on clinical risk factors. 

The role of para-aortic lymph node dissection in the staging of endometrial carcinoma is debatable. At our center, the decision to perform systematic para-aortic nodal dissection is largely surgeon dependent. Moreover, the necessity of systematic para-aortic lymphadenectomy is being challenged by some surgeons as they believe it increases morbidity without added benefit. Notably, lymphatic drainage of uterine lesions confined to the corpus is primarily to the external iliac and the obturator lymph nodes [8]. In advanced disease, para-aortic nodal involvement may occur via spread through the common iliac lymphatic channels [8]. As such, para-aortic involvement often follows pelvic nodal involvement. Abu-Rustum et al. examined the incidence of isolated para-aortic nodal metastasis in the setting of negative pelvic lymph nodes and found it was approximately 1% in both low and high-grade diseases [9]. In our study, 6 of 118 patients (5.08%) in the PPALN group had positive para-aortic nodal metastasis with negative pelvic lymph nodes. 

The therapeutic effects of lymphadenectomy are an issue of great debate in the gynecologic oncology literature. Findings from two large prospective randomized trials of pelvic lymphadenectomy failed to demonstrate a clear therapeutic benefit [10, 11]. Conversely, Mariani et al. showed that patients with poorly differentiated endometrial adenocarcinoma who underwent retroperitoneal lymphadenectomy had an associated survival advantage [12]. However, this advantage did not extend to the addition of para-aortic lymphadenectomy to the lymph node dissection [12]. Recently, the survival effect of para-aortic lymphadenectomy in endometrial cancer (SEPAL) study aimed to examine whether complete, systematic para-aortic lymphadenectomy would have a survival effect in patients with intermediate and high-risk endometrial carcinomas [13]. The results of this retrospective cohort study showed an increased overall survival in patients who had both pelvic and para-aortic lymph node dissection compared to patients who underwent pelvic lymphadenectomy alone. Notably, the average number of lymph nodes in this study was 34 nodes in patients who had pelvic lymph node dissection and 59 nodes in patients who had pelvic and para-aortic lymph nodes dissection with an average of 24 para-aortic nodes [13]. These numbers are significantly higher than the average nodal dissection quoted in most studies. 

Interestingly, our results indicate that patients in the PPALN group had an increased disease recurrence compared to patients in the PLN group. The number of para-aortic lymph nodes retrieved at dissection was a significant variable in predicting DFS. Abu-Rustum et al. showed that removal of 10 or more regional lymph nodes was indicative of adequate surgical staging [14]. Furthermore, Chan et al. noted an improved DFS in patients with intermediate and high-risk diseases who underwent extensive lymph node dissection [15]. These data show that patients with 10 or more para-aortic nodes had improved DFS compared to those who had less than 10 nodes removed. Furthermore, patients in the PPALN group who had 10 or more para-aortic nodes had similar DFS to patients in the PLN group, while those with less than 10 nodes had a worse DFS than patients in the PLN group. These data suggest that limited para-aortic nodal sampling may not provide survival advantage and may negatively impact DFS. 

Adjuvant treatment is an important consideration in the management of women with endometrial carcinoma. The SEPAL study indicated that adjuvant chemotherapy improves survival in intermediate and high-risk diseases [13]. The majority of these cancers are comprised of aggressive histopathological types including high-grade endometrioid, clear cell, and serous carcinomas. It is well established that clear cell and serous endometrial carcinomas are highly malignant, estrogen-independent tumors and are thus classified as type 2 carcinomas [16, 17]. These subtypes account for 10% of endometrial malignancies but are responsible for approximately 50% of relapses [16, 17]. Similarly, high-grade endometrioid cancers often have an aggressive clinical course. Voss et al. examined the immunohistochemical patterns of grade 3 endometrioid carcinoma and found them to be similar to those of clear cell and papillary serous carcinomas [18]. The authors concluded that grade 3 endometrioid cancer may be better characterized as type 2 cancer and should be treated with similar adjuvant therapy to serous and clear cell carcinoma [18]. Given the aggressive tumor biology of type 2 carcinoma, some authorities believe patients should be managed with a limited staging procedure followed by systemic therapy irrespective of stage. In our series, patients in the PLN group were more likely to receive systemic therapy as compared to patients of similar stage in the PPALN group. Given the presumed comprehensive surgical staging, patients in the PPALN group were less likely to receive comprehensive adjuvant therapy consisting of vaginal cuff brachytherapy, pelvic radiation, and systemic chemotherapy. 

Patients in the PPALN group experienced a decreased DFS than patients in the PLN group. Recurrences in the vagina, pelvis, pelvic lymph nodes, para-aortic lymph nodes, and extraperitoneal sites were similar between the groups. Interestingly, the absence of a para-aortic lymph node dissection in the PLN group did not impact the risk of para-aortic recurrence. Isolated para-aortic lymph node recurrence usually occurs in approximately 6% of women with endometrial carcinoma [8]. Our results revealed 17 patients (6.6%) with para-aortic recurrence-5 in the PLN group (3.59%) and 12 in the PPALN group (10.16%) (*P* = 0.39). Importantly, patients who experienced disease recurrence were successfully salvaged as the OS was similar between the study groups. 

The limitations of this study are inherent to its retrospective nature. Patients underwent surgical staging with or without para-aortic lymph node dissection based on recommendations by the attending surgeon. This decision may have been influenced by preoperative biopsy results, medical or surgical co-morbidities, and surgeon preferences and practice. Patients in the PLN group were older, and tumors in that group were less likely to invade the outer myometrium or the lymphovascular space. To control for the heterogeneity between the groups, multivariate statistical analyses were preformed. Importantly, the heterogeneous variables had no impact on DFS or OS. As such, the results were statistically significant and consequently have clinical relevance. 

In conclusion, patients in the PLN group had improved DFS than patients in the PPALN group. DFS was equivalent between patients in the PLN group and patients in the PALN group who had more than 10 para-aortic lymph nodes removed. Notably, intermediate and high-risk endometrial malignancies often exhibit aggressive tumor biology and may require adjuvant therapy to decrease the risk of recurrence. Importantly, patients in the PLN group were more likely to receive multimodality adjuvant therapy than patients in the PALN group, which may have contributed to their improved survival. Thus, operative staging with pelvic lymphadenectomy alone followed by adjuvant radiation and chemotherapy may represent a safe and effective treatment option for women with this disease. Alternatively, if systematic pelvic and para-aortic lymphadenectomy is performed, thorough nodal dissection is advocated with the goal of obtaining 10 or more nodes per lymphatic chain. If less than 10 para-aortic lymph nodes are sampled, the dissection may be an inadequate triage tool for adjuvant therapy. Hence, adjuvant radiation therapy and chemotherapy should be considered to improve DFS. 

## Supplementary Material

Supplemental Table 1a: Variations of adjuvant therapy administration between the PPALN and the PLN group. Supplemental Table 1b: Brachytherapy, pelvic radiation therapy or chemotherapy are not independent predictors of disease free survival. Supplemental Table 1c: Brachytherapy, pelvic radiation therapy or chemotherapy are not independent predictors of overall survival.

## Figures and Tables

**Table 1:** Demographic and clinical characteristics of patients in the PPALN and the PLN groups.

	PPALN* *N* = 118	PLN** *N* = 139	*P* value
Age			
Mean (SD)	63.1 (10.7)	67.1 (9.5)	0.002
Histology			
Grade 3 endometrioid	52 (44.4%)	33 (23.7%)	0.002
Papillary serous	23 (19.7%)	45 (32.4%)	
Clear cell	9 (7.7%)	15 (10.8%)	
Grade 2 endometrioid	4 (3.4%)	2 (1.4%)	
Mixed	25 (21.4%)	43 (30.9%)	
Stage			
I	66 (55.9%)	74 (53.2%)	0.33
II	7 (5.9%)	12 (8.6%)	
III	35 (29.7%)	33 (23.7%)	
IV	10 (8.5%)	20 (14.4%)	
Lymphovascular invasion			
No	52 (47.7%)	83 (64.8%)	0.008
Yes	57 (52.3%)	45 (35.2%)	
Myometrial invasion			
No	60 (52.6%)	88 (64.7%)	0.05
Yes	54 (47.4%)	48 (35.3%)	
Intraoperative complications			
None	99 (86.1%)	124 (89.9%)	0.44
1 or more	16 (13.9%)	14 (10.1%)	
Postoperative complications			
None	54 (46.6%)	79 (57.2%)	0.09
1 or more	62 (53.4%)	59 (42.8%)	

*Pelvic and para-aortic lymph node group.

**Pelvic lymph node group.

**Table 2:** Disease-free survival analysis adjusting for the following variables: tumor histology, lymphovascular invasion, myometrial invasion, and number of para-aortic lymph nodes.

	No recurrence *N* = 183	Recurrence *N* = 74	Age-adjusted HR (95% CI)	Fully adjusted* HR (95% CI)	*P*
Histology					
Endometrioid/mixed	112 (61.5%)	52 (70.3%)	1.00	1.00	
Clear cell	17 (9.3%)	7 (9.5%)	0.95 (0.42, 2.14)	1.33 (0.58, 3.05)	0.50
Papillary serous	53 (29.1%)	15 (20.3%)	0.64 (0.36, 1.15)	0.68 (0.37, 1.26)	0.23
Lymphovascular invasion					
No	112 (67.1%)	23 (32.9%)	1.00	1.00	
Yes	55 (32.9%)	47 (67.1%)	2.99 (1.82, 4.93)	1.67 (0.91, 3.07)	0.10
Myometrial invasion					
No	121 (67.6%)	27 (38.0%)	1.00	1.00	
Yes	58 (32.4%)	44 (62.0%)	2.76 (1.70, 4.45)	1.69 (0.93, 3.06)	0.08
Lymph nodes					
PLN	111 (60.7%)	28 (37.8%)	1.00	1.00	
PPALN < 10*	56 (30.6%)	42 (56.8%)	2.16 (1.33, 3.52)	2.34 (1.36, 4.02)	0.002
PPALN ≥ 10**	16 (8.7%)	4 (5.4%)	1.06 (0.37, 3.01)	1.36 (0.44, 4.24)	0.59

*PPALN patients with less than 10 para-aortic nodes dissected.

**PPALN patients with 10 or more dissected para-aortic nodes.

**Table:** (a)

	PPALN			PLN
	All	<10	≥10	
Positive pelvic lymph nodes				
None	84 (71.2)	68 (69.4)	16 (80.0)	98 (70.5)
1 or more	34 (28.8)	30 (30.6)	4 (20.0)	41 (29.5)
Mean (SD)	1.0 (2.4)	1.1 (2.6)	0.4 (1.1)	0.5 (1.1)
				
Positive para-aortic lymph nodes				
None	92 (78.0)	75 (76.5)	17 (85.0)	—
1 or more	26 (22.0)	23 (23.5)	3 (15.0)	—
Mean (SD)	0.4 (0.9)	0.4 (1.0)	0.2 (0.4)	—

**Table:** (b)

PPALN (*P* < 0.0001)	Negative pelvic and para-aortic nodes	Positive pelvic and para-aortic nodes	Positive pelvic nodes only	Positive para-aortic nodes only
118 (100)	78 (66.1)	20 (16.9)	14 (11.8)	6 (5.08)

**Table:** (a)

	PPALN *N* = 118	PLN *N* = 139	Chi-square *P* value
Vagina			
No	39 (84.8%)	24 (85.7%)	0.91
Yes	7 (15.2%)	4 (14.3%)	
Pelvic lymph node			
No	38 (82.6%)	24 (85.7%)	0.72
Yes	8 (17.4%)	4 (14.3%)	
Pelvis			
No	34 (73.9%)	22 (78.6%)	0.65
Yes	12 (26.1%)	6 (21.4%)	
Para-aortic lymph node			
No	33 (71.7%)	23 (82.1%)	0.31
Yes	13 (28.3%)	5 (17.9%)	
Extraperitoneal			
No	21 (45.7%)	12 (42.9%)	0.81
Yes	25 (54.3%)	16 (57.1%)	
Abdomen			
No	33 (71.7%)	13 (46.4%)	0.03
Yes	13 (28.3%)	15 (53.6%)	

**Table:** (b)

	Alive *N* = 63	Dead *N* = 11	Age-adjusted HR (95% CI)	Fully adjusted* HR (95% CI)	*P*
Vagina					
No	52 (82.5%)	11 (100.0%)			
Yes	11 (17.5%)	0 (0%)			
Pelvic lymph node					
No	53 (84.1%)	9 (81.8%)	1.00	1.00	
Yes	10 (15.9%)	2 (18.2%)	0.64 (0.12, 3.31)	0.22 (0.02, 2.43)	0.22
Pelvis					
No	48 (76.2%)	8 (72.7%)	1.00	1.00	
Yes	15 (23.8%)	3 (27.3%)	1.06 (0.28, 4.02)	1.41 (0.15, 13.1)	0.76
Para-aortic lymph node					
No	48 (76.2%)	8 (72.7%)	1.00	1.00	
Yes	15 (23.8%)	3 (27.3%)	0.46 (0.11, 1.93)	0.37 (0.04, 3.16)	0.36
Extraperitoneal					
No	31 (49.2%)	2 (18.2%)	1.00	1.00	
Yes	32 (50.8%)	9 (81.8%)	3.26 (0.69, 15.4)	10.9 (0.42, 285)	0.15
Abdomen					
No	39 (61.9%)	7 (63.6%)	1.00	1.00	
Yes	24 (38.1%)	4 (36.4%)	1.46 (0.39, 5.43)	1.19 (0.16, 8.87)	0.86

*Adjusted for age (continuous), year of surgery (continuous), lymph nodes (PLN and PALN), histology (endometrioid/mixed, clear cell, and papillary serous), lymphovascular invasion, and myometrial invasion.
