# Supplementary material for: The Role of Para-Aortic Lymphadenectomy in the Surgical Staging of Women with Intermediate and High-Risk Endometrial Adenocarcinomas
Source: Int J Surg Oncol. 2013 Feb 27;2013:858916. doi: 10.1155/2013/858916 (PMC3600173; doi:10.1155/2013/858916)
Supplement: Supplementary file 1 — Supplemental Table 1a: Variations of adjuvant therapy administration between the PPALN and the PLN group. Supplemental Table 1b: Brachytherapy, pelvic radiation therapy or chemotherapy are not independent predictors of disease free survival. Supplemental Table 1c: Brachytherapy, pelvic radiation therapy or chemotherapy are not independent predictors of overall survival. [file 858916.f1.pdf]

Supplemental Table 1

Supplemental Table 1a: Variations of adjuvant therapy administration between the PPALN and the PLN group.

	PPALN N=118	PLN N=139	p-value
Adjuvant therapy			
None	21 (17.8%)	28 (20.1%)	0.02
Vaginal brachytherapy alone	30 (25.4%)	16 (11.5%)	
Pelvic XRT* alone	3 (2.5%)	3 (2.2%)	
Pelvic XRT and brachytherapy	8 (6.8%)	12 (8.6%)	
Chemotherapy alone	18 (15.3%)	14 (10.1%)	
Chemotherapy + brachytherapy	14 (11.9%)	13 (9.4%)	
Chemotherapy + XRT	3 (2.5%)	13 (9.4%)	
Chemotherpy + brachytherapy + XRT	21 (17.8%)	40 (28.8%)	

* Radiation therapy

Supplemental Table 1b: Brachytherapy, pelvic radiation therapy or chemotherapy are not independent predictors of disease free survival.

	No recurrence N=183	Recurrence N=74	Age adjusted HR (95% CI)	Fully adjusted* HR (95% CI)	p
Adjuvant therapy					
None	39 (21.3%)	10 (13.5%)	1.00	1.00	
Brachytherapy and/or Pelvic radiation therapy	59 (32.2%)	13 (17.6%)	0.82 (0.36, 1.90)	0.96 (0.40, 2.32)	0.93
Chemotherapy	85 (46.4%)	51 (68.9%)	1.83 (0.92, 3.66)	1.73 (0.82, 3.69)	0.15

Supplemental Table 1c: Brachytherapy, pelvic radiation therapy or chemotherapy are not independent predictors of overall survival.

	Alive N=244	Dead N=13	Age adjusted HR (95% CI)	Fully adjusted* HR (95% CI)	p
Adjuvant therapy					
None	47 (19.3%)	2 (15.4%)	1.00	1.00	
Brachytherapy and/or radiation	71 (29.1%)	1 (7.7%)	0.28 (0.02, 3.05)	0.74 (0.04, 13.4)	0.84
Any chemotherapy	126 (51.6%)	10 (76.9%)	1.65 (0.36, 7.63)	1.66 (0.17, 15.8)	0.66
